# Evaluating Novel Biomarkers for Personalized Medicine

**DOI:** 10.3390/diagnostics14060587

**Published:** 2024-03-11

**Authors:** Tudor Drugan, Daniel Leucuța

**Affiliations:** Department of Medical Informatics and Biostatistics, Faculty of Medicine, Iuliu Hațieganu University of Medicine and Pharmacy, 400349 Cluj-Napoca, Romania

## 1. Introduction

Personalized medicine, sometimes referred to as precision medicine, is a paradigm shift in healthcare. This model is based on the concept of customizing prevention and treatment approaches to particular cohorts of individuals, drawing on the genetic predispositions, lifestyle choices, and distinctive personal circumstances of individuals. Therefore, uniform therapeutic modalities are not implemented across patient populations as they are with conventional therapies; rather, individual patient characterization and comprehensive pre-testing are emphasized to discern the optimal treatment avenue. Remarkably, traditional one-size-fits-all therapeutic concepts often ignore critical individual variables such as genetic makeup, health status, age, and gender, hence resulting in variable treatment outcomes, from remarkable efficacy to complete inefficiency.

Modern medical research has been increasingly devoted to pioneering individualized diagnostic methodologies and pharmacotherapies personalized to the distinct needs of each patient. Within this context, biomarkers specific to disease provide useful information about the type, molecular etiology, and stage of a disease, leading the way for personalized therapeutic intervention [1,2]. A biomarker can be measured quantitatively and is indicated according to objective evidence representing a biological process, the stage of a disease, or the response of an organism to a given therapeutic intervention.

These biomarkers include a wide array of biological substances or characteristics, such as molecules, nucleic acids (DNA—Deoxyribonucleic Acid, mRNA—Messenger Ribonucleic Acid), microRNA, small interfering RNA, proteins, proteoglycans, lipids, sphingolipids, cells, and imaging features that are detectable and quantifiable in biological samples such as blood, urine, tissues, or imaging scans [3,4]. 

In assessing patients’ health, biomarkers play a crucial role in several aspects:

- Disease Diagnosis: Biomarkers can assist in the early recognition and diagnosis of disease, mainly in targeting molecular signatures or definite abnormalities that are linked to a condition. For example, high levels of certain proteins in the blood could signify cancer is present in the body.
- Prognosis of Disease: Biomarkers may also become useful information tools for prognosis, that is, predicting how a disease is likely to develop or run its course. This is able to help physicians estimate how the disease will progress over time, the risk of it recurring, and overall survival rates, which all help to manage patients and aid in the prescription of treatments.
- Choice of Treatment: Biomarkers can help to decide who may best be treated using a particular therapy. Biomarker-guided therapy can hence select the right treatment for the right patients based on individual characteristics so as to optimize the treatment efficacy and minimize any adverse effects.
- Biomarker assessment can be used to monitor the response to treatment to longitudinally capture its course and any deviations that occur in biological markers associated with disease progression or the therapeutic response from baseline to the final round. In general, the use of biomarkers is increasing across the board and within different medical disciplines to further develop personalized medicine and deliver precision healthcare.

The assessment of biomarkers in medical studies is systematically undertaken to estimate validity, reliability, and clinical utility in light of providing an appropriate diagnosis, prognosis, or prediction or following diagnosis progress. In terms of the process, biomarker assessment generally includes some essential steps:

- Biomarker identification and selection: Relevant biomarker candidates are identified based on preclinical studies, exploratory analyses, and literature reviews and should be related to the indication of these studies, if relevant. A biomarker is a molecular, cellular, or imaging-based characteristic that facilitates distinguishing between the different stages of a disease and predicting the impact of therapeutic interventions.
- Analytical validation: This is how the technical performance characteristics of an assay measuring biomarkers or of the method of measurement itself are examined. Here, the parameters evaluated are the sensitivity, specificity, accuracy, precision, reproducibility, and robustness of biomarker measurement to ensure its reliability across laboratories and platforms.
- Validation of clinical utility: In addition to defining diagnostic or prognostic accuracy, validation of a biomarker includes evaluation of its clinical utility, where clinical utility means the impact of using a biomarker on patient management decisions, therapeutic outcomes, and healthcare resource utilization.
- Long-term monitoring and further research: Following regulatory approval and adoption into practice, monitoring and further research will have to continue with a view to realizing in real-life settings what can be achieved using biomarker-guided interventions, including their long-term safety and effectiveness. Finally, the execution of follow-up post-marketing surveillance, longitudinal observational studies, and comparative effective research aimed at refining clinical algorithms and optimizing the patient outcomes may be required.

In summary, assessing biomarkers in medical research involves a complex procedure demanding rigorous scientific examination, clinical verification, and regulatory supervision to guarantee their dependable and purposeful utilization in healthcare environments. Proficient biomarker assessment has the potential to expedite the advancement of personalized medicine methodologies, enhance clinical decision-making, and ultimately elevate the quality of patient care.

## 2. Brief Overview and List of Contributions

The Special Issue of the MDPI scientific journal *Diagnostics*, entitled “Evaluating Novel Biomarkers for Personalized Medicine”, will focus on this exact topic. Indeed, the eleven articles in this issue cover a wide array of medical subfields, with a scope intended to be widely applicable and demonstrative of the great importance of the research on biomarkers in personalized healthcare. From predicting the treatment outcomes in cancer to assessing inflammatory status in cardiovascular disease, important insights into how biomarkers may transform clinical practice are provided.

For instance, the use of the newly developed GLUCAR Index is assessed for the prediction of the risk of teeth extraction among nasopharyngeal cancer patients receiving chemoradiotherapy and of the inflammatory status of patients who have undergone transcatheter aortic valve replacement due to symptomatic aortic stenosis using machine learning techniques.

Further, the action of serum adropin levels in kidney transplant recipients; the prognostic markers in patients suffering from chronic obstructive pulmonary disease and COVID-19; and the effect of triiodothyronine and protein malnutrition on pulse wave velocity in pre-dialysis patients with chronic kidney disease are all analyzed.

Furthermore, it includes pioneering research on the detection of optical coherence tomography markers for multiple sclerosis diagnosis and management, histopathological biomarkers in coronavirus disease fatalities, and the value of immuno-histochemistry in determining the prognosis for different liver cancers.

Lastly, discussed as newly developed biomarkers are chitotriosidase and neopterin for the prognosis in gastric cancer; systemic inflammatory markers and their interaction with glucose transporter expression in non-small-cell lung carcinoma; and salivary biomarkers of anti-epileptic drugs.

These articles therefore indicate the evolving status of the research on biomarkers and its vital involvement in driving personalized medicine, ultimately leading to much more focused and effective patient care strategies.

## 3. Conclusions

In conclusion, biomarkers greatly contribute to correctly assessing a patient’s status in personalized medicine, as they provide invaluable insight into individual biological characteristics and the process of disease. Thus, according to the careful estimation of biomarkers in individuals, healthcare providers are able to design treatment strategies, optimize the therapeutical outcomes, and eventually provide improved patient care within a new era of personalized medicine.
